# RSV infection-elicited high MMP-12–producing macrophages exacerbate allergic airway inflammation with neutrophil infiltration

**DOI:** 10.1016/j.isci.2021.103201

**Published:** 2021-10-02

**Authors:** Airi Makino, Takehiko Shibata, Mashiro Nagayasu, Ikuo Hosoya, Toshiyo Nishimura, Chihiro Nakano, Kisaburo Nagata, Toshihiro Ito, Yoshimasa Takahashi, Shigeki Nakamura

**Affiliations:** 1 Department of Microbiology, Tokyo Medical University, Tokyo 160-8402, Japan; 2 Department of Immunology, National Institute of Infectious Diseases, Tokyo 162-8640, Japan; 3 Department of Biomolecular Science, Faculty of Science, Toho University, Chiba 274-8510, Japan; 4 Graduate School of Health Care Science, Bunkyo Gakuin University, Tokyo 113-8668, Japan; 5 Department of Immunology, Nara Medical University, Kashihara, Nara 634-8521, Japan; 6 Division of Respiratory Medicine, Department of Internal Medicine, Toho University Ohashi Medical Center, Tokyo 153-0044, Japan

**Keywords:** Pathophysiology, Immunology, Virology, Cell biology

## Abstract

Respiratory syncytial virus (RSV) infection often exacerbates bronchial asthma, but there is no licensed RSV vaccine or specific treatments. Here we show that RSV-induced alveolar macrophages, which produce high levels of matrix metalloproteinase-12 (MMP-12), exacerbate allergic airway inflammation with increased neutrophil infiltration. When mice subjected to allergic airway inflammation via exposure to the house dust mite antigen (HDM) were infected with RSV (HDM/RSV), MMP-12 expression, viral load, neutrophil infiltration, and airway hyperresponsiveness (AHR) were increased compared to those in the HDM and RSV groups. These exacerbations in the HDM/RSV group were attenuated in MMP-12-deficient mice and mice treated with MMP408, a selective MMP-12 inhibitor, but not in mice treated with dexamethasone. Finally, M2-like macrophages produced MMP-12, and its production was promoted by increase of IFN-β-induced IL-4 receptor expression with RSV infection. Thus, targeting MMP-12 represents a potentially novel therapeutic strategy for the exacerbation of asthma.

## Highlights

• RSV–induced MMP-12 exacerbates allergic airway inflammation
• RSV–elicited IL-4Rα^high^ M2-like macrophages highly express MMP-12
• MMP-12 enhances neutrophil infiltration via promotion of IL-17A and CXCL1 production
• MMP-12 inhibitor attenuates RSV-induced neutrophilic airway inflammation

## Graphical abstract

**Figure:**
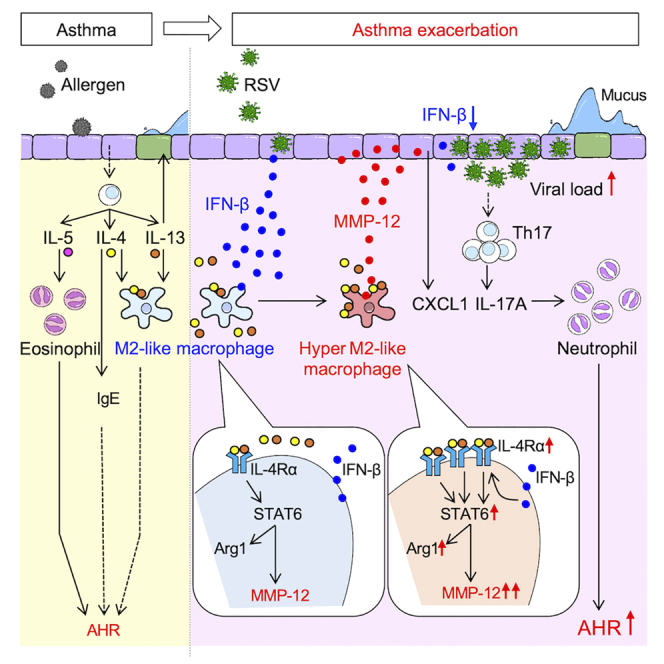

## Introduction

Asthma is a chronic airway inflammatory disease characterized by wheezing and increasing airway hyperresponsiveness (AHR) with allergic airway inflammation that promotes persistent physiological and structural remodeling events in the lungs (Galli et al., 2008). Several environmental allergens and/or viruses are known to trigger and exacerbate asthma via their effects on innate and adaptive immune cells (Jackson et al., 2011; O'Byrne, 2011). For example, dendritic cells in the airways induce sensitization to allergens, leading to the development of IL-4-, IL-5-, and IL-13-induced Th2-dependent airway inflammation (Lambrecht et al., 2000; van Rijt et al., 2005). Th2-type cytokines, such as IL-4 and IL-13, are involved in the differentiation of alternatively activated (M2)-like macrophages (Lambrecht et al., 2000), which represent a source of airway remodeling factors, such as arginase (Maarsingh et al., 2011).

Thus, asthma has traditionally been viewed as a Th2 disease with increased IgE levels, eosinophilic inflammation, and M2 macrophages contributing to AHR (Robinson et al., 1992), whereas the findings in an asthma mouse model suggested that Th1 cells and neutrophils also modulate the disease (Cui et al., 2005). In addition, Th17 cells and group 3 innate lymphoid cells (ILC3s) have been identified to contribute to airway inflammation and AHR (Cosmi et al., 2011; Hekking et al., 2018; Kim et al., 2014). Hence, recent attention has also been directed at characterizing factors that modulate immune cell responses in response to environmental allergens.

However, treatments centered on inhaled steroids and long-acting inhaled β2 agonists are currently used. In the case of severe asthma, oral steroids are used as a therapeutic approach, but the symptoms sometimes cannot be properly managed (Simpson et al., 2014; Uddin et al., 2010, 2013). Exacerbation of asthma results in reduced lung function, markedly reduced quality of life, and financial burdens. The exacerbation is often caused by viral or bacterial infections, among which respiratory syncytial virus (RSV) is associated with exacerbations in patients of various ages with asthma (Busse et al., 2010; Hassanzad et al., 2019; Jartti and Gern, 2017; Zheng et al., 2018). However, much remains unknown about the mechanism of exacerbation of asthma caused by RSV infection. In addition, no therapeutic drug targeting the exacerbation of asthma has been put into practical use.

RSV infects almost 100% of infants by 2 years of age, and it is involved in the development of respiratory diseases, including exacerbation of pneumonia and asthma (Falsey et al., 2006). The prevention of RSV infection has attracted attention, but this goal is difficult to attain because of the lack of an effective and safe vaccine against RSV. Therefore, it is important to clarify the mechanism by which RSV infection exacerbates pneumonia and asthma to facilitate the development of novel treatments. Regarding this issue, we recently identified one mechanism by which RSV infection exacerbates bacterial pneumonia (Shibata et al., 2020). Thus, in this study, we attempted to clarify the mechanism by which RSV infection exacerbates asthma using mouse models.

## Results

### MMP-12 levels were increased during allergic airway inflammation after RSV infection

**Figure 1:**
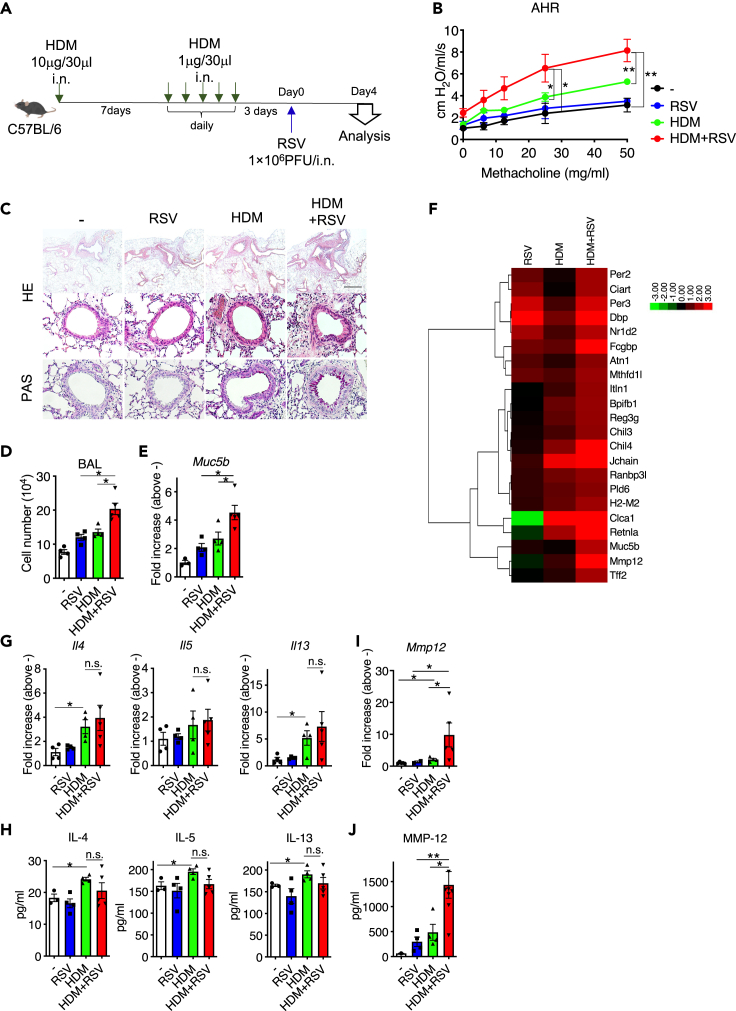
Matrix metalloproteinase-12 (MMP-12) levels are increased by allergic airway inflammation after respiratory syncytial virus (RSV) infection (A) Mice with house dust mite antigen (HDM)-induced allergic airway inflammation were infected with RSV. (B) Measurement of respiratory system resistance (Rrs) for airway hyperresponsiveness (AHR) in vehicle-injected mice (–: control), RSV-infected mice (RSV), HDM-sensitized mice (HDM), and RSV-infected HDM-sensitized mice (HDM/RSV). (C) Hematoxylin and eosin (HE) staining and periodic acid-Schiff staining (PAS) in lung tissue in the control, RSV, HDM, and HDM/RSV groups. Scale bars, 200 μm (upper), 50 μm (middle and lower). (D) Number of total cells in the bronchoalveolar lavage (BAL) fluid in control, RSV, HDM, and HDM/RSV groups. (E) mRNA level of *Muc5b* in whole lungs from the control, RSV, HDM, and HDM/RSV groups. (F) Heat map presents the results of DNA microarray in the control, RSV, HDM, and HDM/RSV groups. (G and H) mRNA (G) and protein (H) levels of IL-4, IL-5, and IL-13 in whole lungs. (I and J) mRNA (I) and Protein (J) levels of MMP-12 in whole lungs. The data are expressed as the mean ± SEM (n = 5–6). ∗p < 0.05, ∗∗p < 0.01, n.s., not significant. See also Figure S1.

RSV infection often exacerbates asthma, but the mechanism remains unclear. To reveal the mechanism, we first developed a mouse model of the RSV-induced exacerbation of allergic airway inflammation (Figure 1A). Recurrent HDM injections induced allergic airway inflammation accompanied by increased AHR (Figures 1B and S1), the peribronchial accumulation of inflammatory cells (Hematoxylin and eosin: HE), and goblet cell metaplasia (periodic acid-Schiff staining: PAS) (Figure 1C). Furthermore, RSV infection following HDM exposure significantly exacerbated these allergic airway responses compared to the findings in the HDM and RSV groups. The cell number in the bronchoalveolar lavage (BAL) fluid (Figure 1D) and *Gob5b* transcript expression (Figure 1E) quantitatively show the magnitude of airway infiltration and mucus production, respectively, and these results were consistent with the results of HE and PAS as shown Figure 1C. To reveal the genes related to the findings in the HDM/RSV group, DNA microarray was performed (Figure 1F). Several genes were upregulated by more than 2-fold in the HDM/RSV group compared to the findings in control mice. MMP-12, which showed a characteristic expression pattern, increased in the HDM/RSV group, but not in the HDM and RSV groups. Because the expression of other genes, apart from MMP-12, were also elevated in HDM and RSV groups, we decided to focus on MMP-12 in this study. In fact, MMP-12 is associated with disease severity in patients with asthma (Hinks et al., 2016; Mukhopadhyay et al., 2010), although the mechanism has not been elucidated. Incidentally, asthma is characterized by increased Th2 cytokine production; however, in this model, Th2 cytokines, including IL-4, IL-5, and IL-13, which were upregulated in the HDM group, were not further upregulated in the HDM/RSV group at the mRNA (Figure 1G) and protein (Figure 1H) level. By contrast, the mRNA (Figure 1I) and protein (Figure 1J) expression of MMP-12 was also higher in the HDM/RSV group than in the RSV and HDM groups. Taken together, the increasing MMP-12 production in the airway induced by RSV might be responsible for the exacerbation of AHR following HDM exposure.

### MMP-12 exacerbated allergic airway inflammation

**Figure 2:**
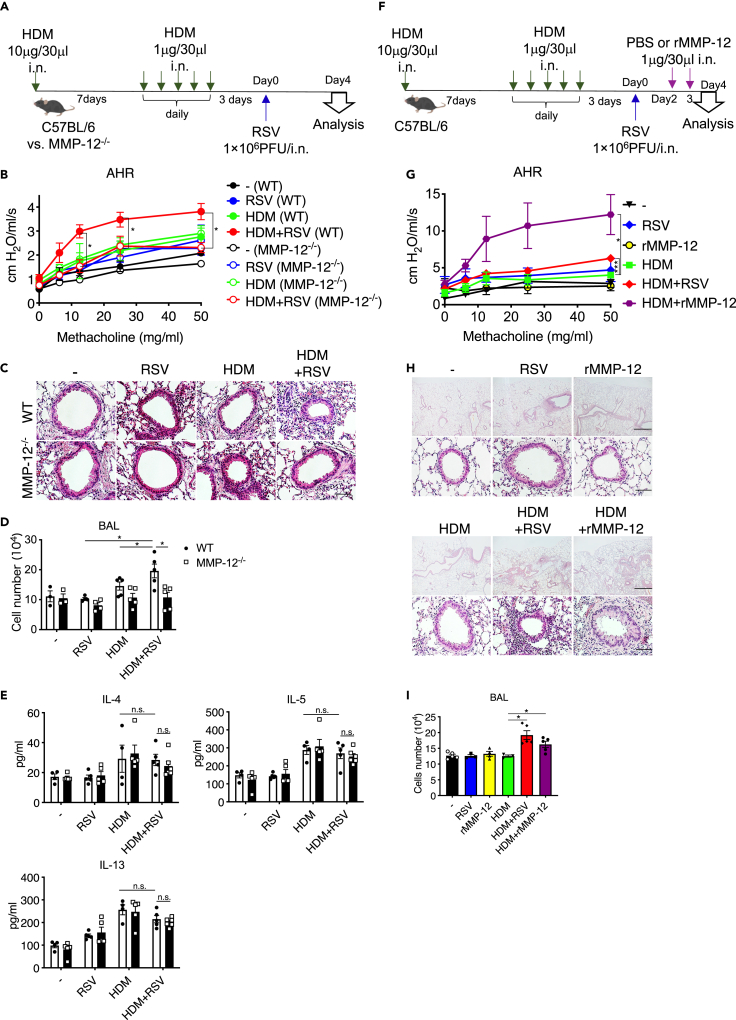
MMP-12 exacerbates allergic airway inflammation (A) Wild-type (WT) or MMP-12 knockout (KO) mice with HDM-induced allergic airway inflammation were infected with RSV. (B) Measurement of AHR in control, RSV, HDM, and HDM/RSV with WT or MMP-12 KO. (C) HE staining of lung tissue in the control, HDM, RSV, and HDM/RSV groups of WT and MMP12 KO mice. Scale bars, 50 μm. (D) Number of total cells in the BAL fluid in control, RSV, HDM, and HDM/RSV groups with WT or MMP-12 KO. (E) Protein levels of IL-4, IL-5, and IL-13 in whole lungs in control, RSV, HDM, and HDM/RSV groups with WT or MMP-12 KO. (F) Mice with HDM-induced allergic airway inflammation were infected with RSV or recombinant MMP-12 (rMMP-12). (G) Measurement of AHR of the control, RSV, rMMP-12, HDM, HDM/RSV, and HDM + rMMP-12 groups. (H) HE staining of lung tissue in the RSV, rMMP-12, HDM, HDM/RSV, and HDM + rMMP-12 groups. Scale bars, 200 μm (upper), 50 μm (lower). (I) Number of total cells in the BAL fluid of the control, RSV, rMMP-12, HDM, HDM/RSV, and HDM + rMMP-12 groups. The data are expressed as the mean ± SEM (n = 4–6). ∗p < 0.05, ∗∗∗p < 0.001.

To reveal whether MMP-12 is involved in the development of the observed pathogenesis in the HDM/RSV group, the responses were compared between wild-type (WT) and MMP-12–knockout (KO) mice (Figure 2A). In the HDM/RSV group, the magnitude of AHR and peribronchial inflammatory cell accumulation was significantly lower in MMP-12 KO mice than in WT mice (Figures 2B and 2C). Consistent with these results of airway inflammation, the number of cells in BAL fluid was also significantly increased in the HDM/RSV of WT compared to RSV and HDM but the increase was significantly suppressed in MMP-12 KO mice (Figure 2D). In contrast, in WT HDM/RSV, Th2 cytokine levels were not increased compared to RSV and HDM groups, and there was no particular change in MMP-12 KO mice (Figure 2E). The results illustrated the involvement of MMP-12, but not Th2 cytokines, in the exacerbation of allergic airway inflammation by RSV infection in our model. Therefore, to confirm the contribution of MMP-12 to the exacerbation, we investigated the responses after the administration of recombinant MMP-12 (rMMP-12) instead of RSV infection in HDM mice (Figure 2F). The administration of rMMP-12 to HDM mice significantly promoted AHR and peribronchial accumulation of inflammatory cells (Figures 2G and 2H). The number of cells in BAL fluid was also significantly increased in rMMP-12-treated HDM mice and HDM/RSV mice (Figure 2I). Thus, MMP-12 strongly contributes to the exacerbation of allergic airway inflammation caused by RSV infection.

### M2-like alveolar macrophages produced MMP-12

**Figure 3:**
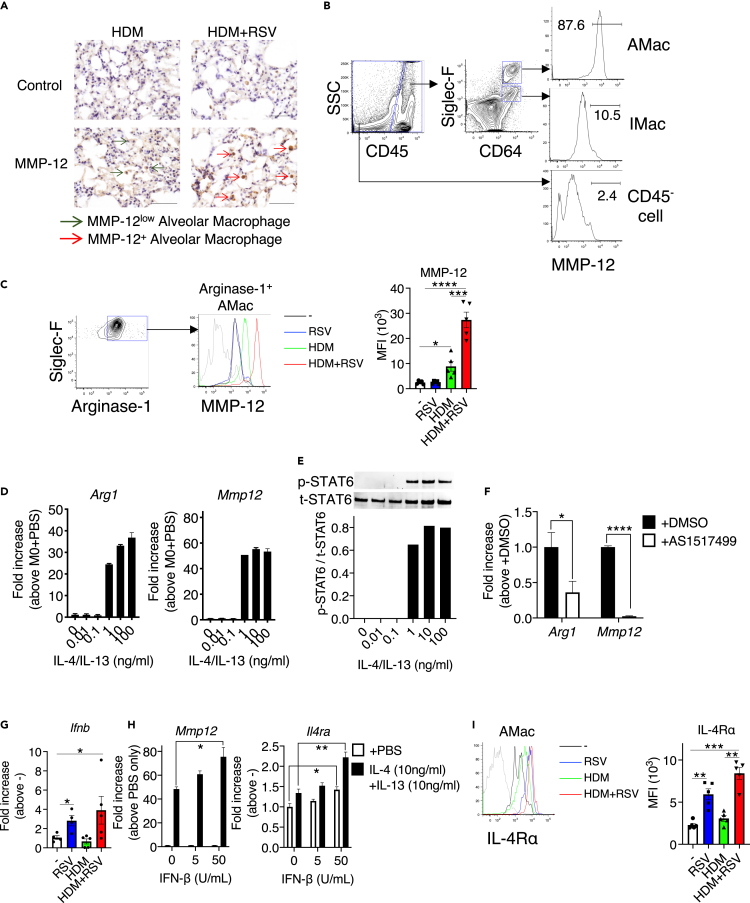
IL-4 receptor α (IL-4Rα)^high^ M2-like alveolar macrophages highly produces MMP-12 (A) Immunohistochemical staining of MMP-12 in lung tissue. Scale bars, 200 μm. (B) Flow cytometry of MMP-12 expression by alveolar macrophages (AMac), interstitial macrophages (IMac), and CD45^−^ cells in HDM mice. CD45^+^Siglec-F^high^CD64^+^: AMac, CD45^+^Siglec-F^+^CD64^+^: IMac. (C) Flow cytometric analysis and median fluorescence intensity of MMP-12 in M2-like alveolar macrophages (arginase-1^+^ macrophages gated on Siglec-F^high^CD64^+^) in the whole lungs of mice in control (black), RSV (blue), HDM (green), and HDM/RSV groups (red). Gray line denotes isotype control. (D) mRNA levels of *Arg1* and *Mmp12 in* RAW264.7 macrophages after stimulation with IL-4 and IL-13 *in vitro*. (E) Activation of STAT6 in RAW264.7 macrophages stimulated with IL-4 and IL-13 for 1 h. The ratios of phosphorylated (p)-STAT6 to total (t)-STAT6 are present. (F) *Arg1* and *Mmp12* mRNA levels in RAW264.7 macrophages treated with AS1517499 (STAT6 inhibitor). (G) mRNA levels of *Ifnb* in whole lungs from mice in the control, RSV, HDM, and HDM/RSV groups. (H) mRNA levels of *Mmp12* and *Il4ra in* RAW264.7 macrophages after stimulation with IFN-β in the presence of IL-4 (10 ng/mL) and IL-13 (10 ng/mL). (I) Flow cytometric analysis of IL-4Rα and the median fluorescence intensity of IL-4Rα and MMP-12 in alveolar macrophages in the whole lungs of mice in the control (black), RSV (blue), HDM (green), and HDM/RSV groups (red). Gray line denotes the isotype control. The data are expressed as the mean ± SEM (n = 5–6 [A, B, F, H], n = 3 [C, D, E, G]). ∗p < 0.05, ∗∗p < 0.05, ∗∗∗p < 0.001, ∗∗∗∗p < 0.0001.

MMP-12 induced by RSV infection in HDM-exposed mice exacerbated the pathogenesis of airway inflammation, including increased AHR. We then attempted to identify the responsible cells that produce MMP-12 and regulate the subsequent responses in HDM/RSV. Immunohistochemical analysis of lung sections revealed the expression of MMP-12 protein by alveolar macrophages in the HDM/RSV group (Figure 3A). MMP-12–positive alveolar macrophages were also detected in HDM mice, but the intensity was weaker than that in HDM/RSV mice. Flow cytometry also revealed that alveolar macrophages expressed MMP-12 more strongly than interstitial macrophages and CD45^−^ structural cells in the lungs (Figure 3B). Importantly, M2-like macrophages, which expressed arginase-1, in HDM/RSV mice expressed MMP-12 more intensely than M2-like macrophages from the other groups, including HDM group (Figure 3C).

We then examined the strong induction of MMP-12 in alveolar macrophages in HDM/RSV mice. MMP-12 was also detected in HDM mice with Th2-base immune responses. Thus, Th2 cytokines were cited as potential inducers of MMP-12. In fact, stimulation with the Th2 cytokines IL-4 and IL-13, which were upregulated by HDM exposure *in vivo* and the inducers of M2-like macrophages (Figure 1C), increased the levels of both arginase-1 and MMP-12 in macrophages *in vitro* (Figure 3D). Although the addition of IL-4/IL-13 activated STAT6 in macrophages (Figure 3E), AS1517499, a selective STAT6 inhibitor, significantly suppressed arginase-1 and MMP-12 expression (Figure 3F). These results suggested that IL-4 and IL-13/STAT6 activation is necessary for the induction of MMP-12.

### IFN-β–elicited IL-4 receptor α (IL-4Rα)^high^ macrophages highly expressed MMP-12

MMP-12 was more strongly produced in HDM/RSV mice than in HDM mice (Figure 3C). We then attempted to elucidate the mechanism of increased MMP-12 production in HDM/RSV mice. Unlike MMP-12, IL-4, and IL-13 were not upregulated by RSV infection in HDM mice, as presented in Figures 1G and 1H. Therefore, it was predicted that some factor other than those Th2 cytokines induced by RSV infection accelerates MMP-12 production. As is well known, RSV infection induced type I IFNs, such as IFN-β (Jewell et al., 2007), in the RSV and HDM/RSV groups (Figure 3G). IFN-β accelerated the expression of MMP-12 in IL-4/IL-13-stimulated macrophages in a concentration-dependent manner (Figure 3H). Although the treatment of macrophages with 10 ng/mL IL-4 and IL-13 increased STAT6 activation and MMP-12 expression to near maximal levels, IFN-β further stimulated MMP-12 production. These results also suggested that a certain IFN-β–induced factor was critical for promoting MMP-12 production. As the ligand levels of Th2 cytokines did not differ in HDM/RSV compared with that in HDM, we focused on and investigated the change in receptor expression. As a result, we demonstrated that IFN-β stimulation increased the expression IL-4Rα in macrophages as well as MMP-12 (Figure 3H). In fact, in RSV and HDM/RSV mice, IL-4Rα expression in alveolar macrophages was higher than that in control and HDM mice (Figure 3I). Taken together, these results suggested that an RSV-evoked IFN-β/IL-4Rα axis further increased the production of MMP-12 during allergic airway inflammation, resulting in the exacerbation of airway inflammation.

### High levels of MMP-12 derived neutrophils in HDM/RSV mice

**Figure 4:**
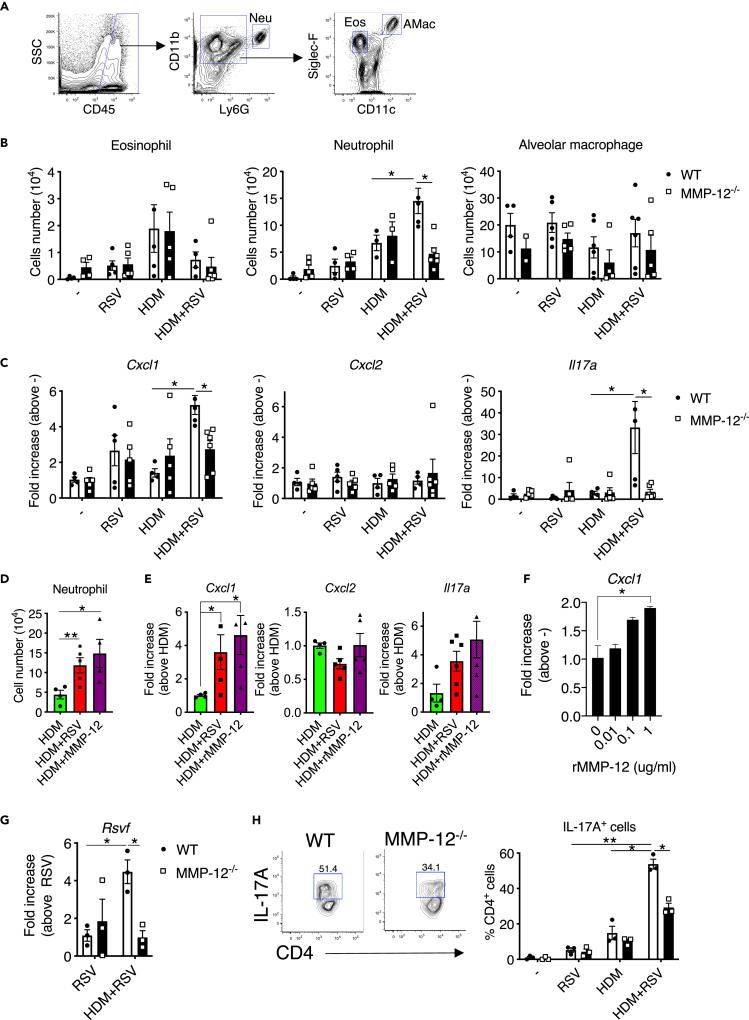
MMP-12 increases neutrophil infiltration during allergic airway inflammation (A) Representative flow cytometric analysis of neutrophils (CD45^+^CD11b^+^Ly6G^high^: Neu), eosinophils (CD45^+^CD11b^+^Siglec-F^+^CD11c^low^: Eos), and alveolar macrophages (CD45^+^CD11b^+^Siglec-F^high^CD11c^high^: AMac) in whole lungs. (B and C) The number of eosinophils, neutrophils, and alveolar macrophages (B) and mRNA levels of *Cxcl1*, *Cxcl2*, and *Il17a* (C) in the control, HDM, RSV, and HDM/RSV groups of WT and MMP12 KO mice. (D and E) Flow cytometric analysis of neutrophils (D) and mRNA levels of *Cxcl1*, *Cxcl2*, and *Il17* (E) in whole lungs from mice in the HDM, HDM/RSV, and HDM + rMMP-12 groups. (F) mRNA levels of *Cxcl1* in lung epithelial cells (MLE-12) stimulated with rMMP-12. (G) Virus titer in the lung of control, HDM, RSV, and HDM/RSV groups of WT and MMP12 KO mice. (H) Cells generated from the lungs of control, HDM, RSV, and HDM/RSV groups of WT and MMP12 KO mice were stimulated with PMA and Ionomycin and then stained for IL-17A. CD4^+^/IL-17A^+^ cells after gating on CD45^+^/CD3^+^ cells in HDM/RSV (left panel). A percentage of IL-17A^+^ cells in CD4^+^ T cells (right panel). The data are expressed as the mean ± SEM (n = 4–6). ∗p < 0.05, ∗∗p < 0.01. See also Figure S2.

As the above analysis demonstrates the mechanism of increased MMP-12 production that exacerbated the allergic airway inflammation in HDM/RSV, we next investigated the mechanism by which high levels of MMP-12 exacerbate allergic airway inflammation. Focusing on the number of inflammatory cells, such as eosinophils, neutrophils, and macrophages in the lung (Figure 4A), in the HDM group, the increase in the number of eosinophils was similar to that in WT and MMP-12 KO mice (Figure 4B). Contrarily, the number of neutrophils was significantly higher in WT HDM/RSV mice than in HDM mice. Furthermore, the increased neutrophil count in WT mice was significantly attenuated in MMP-12 KO mice. Similar to the number of neutrophils, the expression of chemokines and neutrophil activators, especially CXCL1 (KC) and IL-17A, but not CXCL2 (MIP-2), was significantly higher in WT HDM/RSV mice than in MMP-12 KO mice (Figure 4C). In addition, the administration of rMMP-12 instead of RSV infection to HDM mice significantly promoted peribronchial accumulation of neutrophils and mRNA expression of CXCL1 and IL-17A compared to the findings observed in untreated HDM mice (Figures 4D and 4E).

These results suggest that MMP-12 directly or indirectly promotes neutrophil infiltration via increased CXCL1 and IL-17A production in HDM/RSV mice, which led us to investigate the induction mechanism of CXCL1 and IL-17A by MMP-12. We first predicted a direct effect of MMP-12 on lung epithelial cells and/or macrophages. When these cells were treated with rMMP-12 *in vitro* without RSV exposure, the expression of CXCL1, but not CXCL2 and IL-17A, was increased in lung epithelial cells in a concentration-dependent manner (Figure 4F). By contrast, CXCL1 and IL-17A expression in macrophages was not changed by rMMP-12 treatment (data not shown). These results suggested that MMP-12 stimulates epithelial cells to produce CXCL1, whereas it promotes IL-17A expression indirectly through cells other than epithelial cells and macrophages *in vivo*.

Note that virus titer in the WT lung was significantly increased in the HDM/RSV group compared to that in the RSV group, whereas the virus titer was significantly suppressed in MMP-12 KO mice (Figure 4G). These results suggest that MMP-12 has potential to increase viral load. It has been well known that increased RSV induces Th17 cell-mediated IL-17A production (Mukherjee et al., 2011). Moreover, the changes in viral load showed the same tendency as IL-17A production (Figure 4C). Hence, we investigated the changes in IL-17A-producing cells in each group and their involvement in MMP-12. As a result, IL-17A-producing T cells were significantly increased in the HDM/RSV of WT mice compared to that of the RSV and HDM groups, whereas IL-17A-producing T cells were suppressed in MMP-12 KO mice of the RSV and HDM groups (Figure 4H). Incidentally, the number of IFN-γ-producing T cells and IL-4-producing T cells was not further increased in HDM/RSV group compared to RSV and HDM groups (Figure S2). These results suggested that MMP-12, which is increased in HDM/RSV group, promotes IL-17A production by Th17 cells and enhances neutrophil infiltration.

### MMP-12–derived neutrophils increased AHR

**Figure 5:**
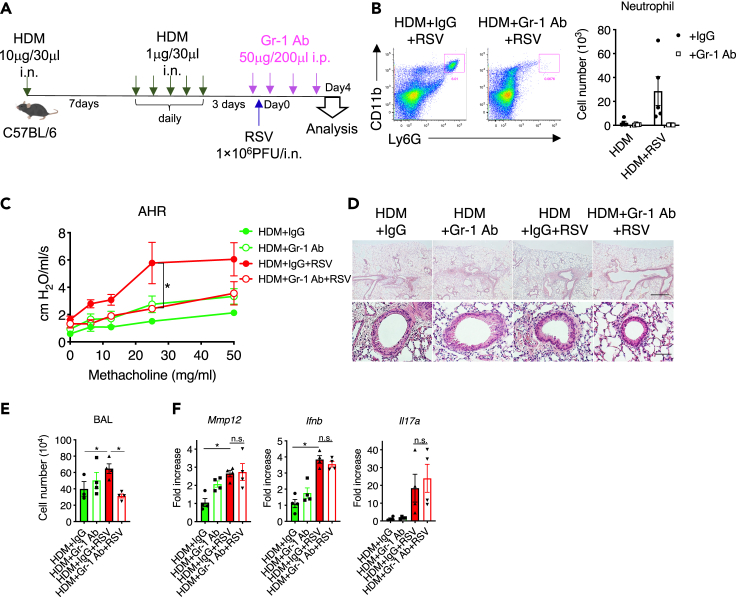
RSV-derived neutrophils exacerbate allergic airway inflammation (A) Anti-Gr-1 antibody (Ab) or control IgG was administered every 24 h for 4 consecutive days starting 2 h before RSV infection. (B) Flow cytometric analysis of neutrophils in whole lungs. CD11b^+^Ly6G^high^: neutrophil. (C) AHR in anti-Gr-1 Ab- or IgG-administered HDM or HDM/RSV mice. (D) HE staining of lung tissue from mice in the HDM + IgG, HDM + Gr-1 Ab, HDM/RSV + IgG, and HDM/RSV + Gr-1 Ab groups. Scale bars, 200 μm (upper), 50 μm (lower). (E) Number of total cells in the BAL fluid from mice in the HDM + IgG, HDM + Gr-1 Ab, HDM/RSV + IgG, and HDM/RSV + Gr-1 Ab groups. (F) mRNA levels of *Mmp12*, *Ifnb*, and *Il17a* in the whole lungs of mice in the HDM + IgG, HDM + Gr-1 Ab, HDM/RSV + IgG, and HDM/RSV + Gr-1 Ab groups. The data are expressed as the mean ± SEM (n = 5–6). ∗p < 0.05.

It appeared that the increase in the number of neutrophils was a hallmark of the HDM/RSV group. To examine whether increases in the number of neutrophils was involved in the exacerbation of AHR induced by HDM exposure, neutrophils were depleted in HDM/RSV mice by injecting anti-Gr-1 Ab (Figure 5A). Ab treatment almost completely depleted neutrophils in the lungs (Figure 5B). When mice in the HDM/RSV group were treated with anti-Gr-1 Ab, AHR, peribronchial inflammatory cell accumulation, and the number of cells in BAL fluid were significantly suppressed compared to the findings in control IgG-treated mice (Figures 5C–5E). These results suggest that increased neutrophil accumulation exacerbates the pathogenesis of HDM after RSV infection. Incidentally, MMP-12, IFN-β, and IL-17A expressions were not changed by anti-Gr-1 Ab treatment in the HDM/RSV group, suggesting that these expressions were not regulated by neutrophils (Figure 5F). Therefore, these upregulations are events that occur before increased neutrophil infiltration.

### MMP408, but not dexamethasone, attenuated airway inflammation in HDM/RSV mice

**Figure 6:**
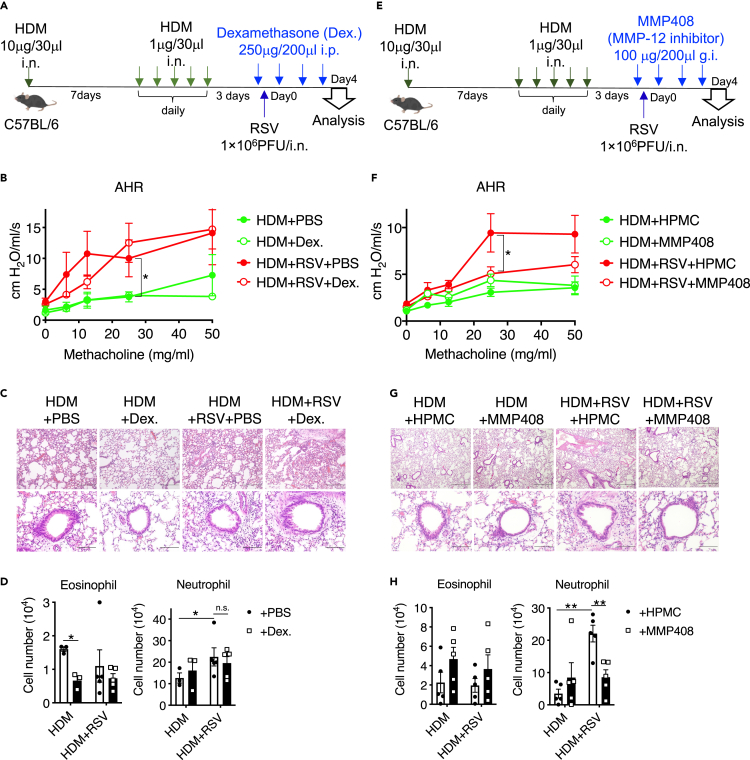
MMP-12 inhibition attenuates RSV-exacerbated allergic airway inflammation (A) Dexamethasone (Dex.) was administered every 24 h for 4 consecutive days starting 2 h before RSV infection. (B) AHR in dexamethasone- or PBS-administered mice in the HDM and HDM/RSV groups. (C) HE staining of lung tissue from mice in the HDM + PBS, HDM + Dex. HDM/RSV + PBS, and HDM/RSV + Dex. groups. Scale bars, 200 μm (upper), 50 μm (lower). (D) Flow cytometric analysis of eosinophils and neutrophils in whole lungs from mice in the HDM + PBS, HDM + Dex. HDM/RSV + PBS, and HDM/RSV + Dex. groups. (E) MMP408 was administered every 24 h for 4 consecutive days starting 2 h before RSV infection. (F) AHR in MMP408- or hydroxypropyl methylcellulose (HPMC)-administered mice in HDM and HDM/RSV groups. (G) HE staining of lung tissue from mice in the HDM + HPMC, HDM + MMP408. HDM/RSV + HPMC, and HDM/RSV + MMP408 groups. Scale bars, 200 μm (upper), 50 μm (lower). (H) Flow cytometric analysis of eosinophils and neutrophils in whole lungs from mice in the HDM + HPMC, HDM + MMP408, HDM/RSV + HPMC, and HDM/RSV + MMP408 groups. The data are expressed as the mean ± SEM (n = 5–6), ∗p < 0.05, ∗∗p < 0.01.

Inhaled steroids are used to treat asthma. To clarify whether steroids are effective in the HDM/RSV model, mice were administered dexamethasone (Figure 6A). As a result, AHR in dexamethasone-treated HDM/RSV mice was not significantly suppressed compared to that in control mice (Figure 6B). Accordingly, the peribronchial accumulation of inflammatory cells was inhibited in dexamethasone-treated HDM mice, but no obvious difference was observed in HDM/RSV mice (Figure 6C). Importantly, the number of eosinophils was significantly decreased after dexamethasone treatment in the HDM group, whereas the number of neutrophils was not decreased by dexamethasone in the HDM or HDM/RSV group (Figure 6D), although reducing neutrophil counts improves airway inflammation in HDM/RSV mice as shown in Figure 5. Instead of dexamethasone, we then examined the effect of MMP-12 inhibition in this model (Figure 6E). Treatment with the MMP-12 inhibitor MMP408 significantly suppressed AHR and the peribronchial accumulation of inflammatory cells in the HDM/RSV group (Figures 6F and 6G). Finally, the number of neutrophils was reduced in MMP408-treated HDM/RSV mice (Figure 6H). Taken together, MMP408 potentially exerts therapeutic effects against the exacerbation of airway inflammation in the HDM/RSV group by decreasing the number of neutrophils.

## Discussion

**Figure 7:**
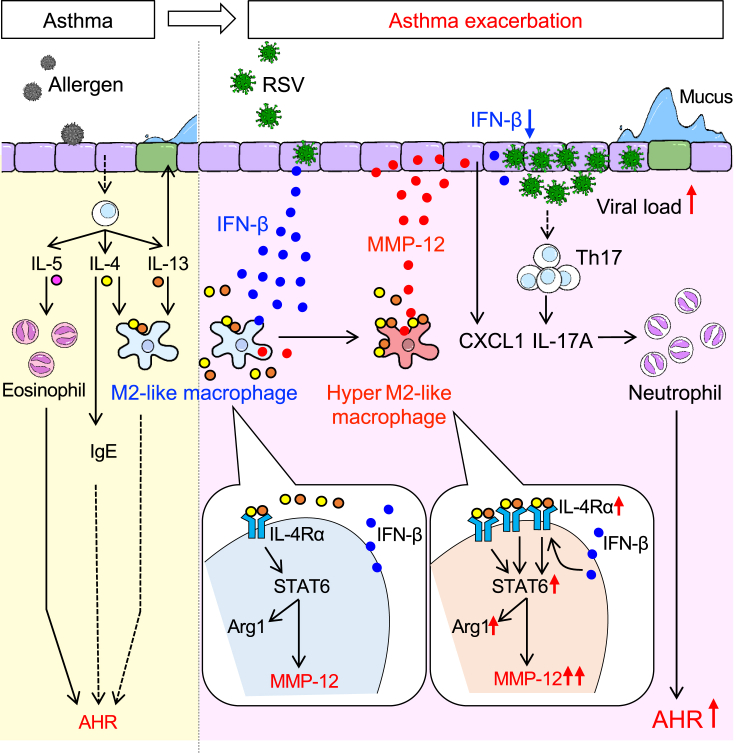
RSV infection-evoked MMP-12 exacerbates allergic airway inflammation with neutrophil infiltration Shortly after RSV infection, the M2-like alveolar macrophages (arginase-1 (Arg1) positive) that highly express IL-4 receptors produce high levels of MMP-12 during allergic airway inflammation. MMP-12 attenuates anti-viral activity and causes the peribronchial accumulation of neutrophils via increased Th17/IL-17A axis. This exacerbates allergic airway inflammation.

In this study, we investigated the mechanism by which RSV infection exacerbates allergic airway inflammation. Our findings revealed for the first time that RSV-evoked MMP-12 exacerbates allergic airway inflammation with neutrophil infiltration (Figure 7). When HDM-sensitized mice were infected with RSV, high levels of MMP-12 were produced by M2-like alveolar macrophages that strongly express IL-4Rα. These high MMP-12 levels accelerated CXCL1 and IL-17A expression and promoted neutrophil infiltration, resulting in increased AHR. Finally, the increased neutrophil counts and AHR in HDM/RSV mice were not attenuated by dexamethasone treatment, whereas the exacerbation was suppressed by the administration of an MMP-12 inhibitor.

Severe RSV infection in early childhood increases the risk of developing severe asthma after growth (Sigurs et al., 2005, 2010). In fact, when 1-week-old mice were infected with RSV and exposed to HDM 5 weeks later (RSV/HDM), AHR in the group was higher than HDM and RSV groups (Figure S3). However, the levels of MMP-12 and neutrophil infiltration were not increased in RSV/HDM compared to HDM group, suggesting that MMP-12/neutrophil found in this study does not apply to the above symptom. Thus, the presence of M2 macrophages, as seen in asthma, is important for the activation of MMP-12/neutrophils axis associated with RSV infection.

MMP-12 is an enzyme mainly produced by macrophages that degrades elastin fiber (Lagente et al., 2009). MMP-12 also exerts immune effects on chemoattractants to monocytes *in vitro*, but the mechanism has not been fully elucidated *in vivo* (Senior et al., 1984). However, we found that MMP-12 increased the production of IL-17A and CXCL1, which promoted neutrophil infiltration *in vivo*. Our findings are important because increased numbers of neutrophils lead to the exacerbation of airway inflammation during asthma (McKinley et al., 2008). Therefore, elucidating the mechanism by which MMP-12 enhances IL-17A and CXCL1 production is important. One of the causes of IL-17A increased expression was the increase in IL-17-producing Th17 cells by MMP-12. MMP-12 degrades type I IFN, which attenuates the anti-viral activity and increases RSV load and neutrophil accumulation into the lungs (Marchant et al., 2014). Furthermore, increased RSV induces Th17 cell-mediated IL-17A production and neutrophilic inflammation (Mukherjee et al., 2011). These may explain the mechanism by which MMP-12 promotes neutrophil infiltration via increased IL-17A production in our model. However, further study is needed to elucidate the mechanism by which MMP-12 induces CXCL1 expression in lung epithelial cells.

Increased MMP-12 production is often observed in chronic obstructive pulmonary disease (COPD) (Churg et al., 2003, 2004; Vlahos et al., 2010). The pathogenesis is attributable to emphysema caused by the decomposition of elastin, which constitutes the alveoli, by MMP-12 (Ohnishi et al., 1998). In our model, when HDM-sensitized mice were infected with RSV, high levels of MMP-12 were produced. Therefore, the development of COPD, particularly emphysema, was expected in HDM/RSV mice, but no such emphysema or elastin degradation was observed. These results suggested that continuous MMP-12 production in the presence of stimuli such as cigarette smoke is probably required for the development of emphysema and that the promotion of neutrophil infiltration rather than structural changes in lung tissue in response to high levels of MMP-12 is an underlying cause of the exacerbation of airway inflammation in our model.

Neutrophilic asthma can be steroid-resistant and severe (Nabe, 2020; Ray and Kolls, 2017), and in our model, resistance to dexamethasone was observed in the HDM/RSV group, which displayed neutrophil infiltration. Importantly, and consistent with the results in MMP-12 KO mice, MMP408 administration attenuated increases of neutrophil infiltration and AHR. In addition, a study in patients with asthma illustrated that MMP-12 gene variant is associated with disease severity, although no attempt has been made to elucidate the mechanism by which MMP-12 is highly expressed (Hinks et al., 2016; Mukhopadhyay et al., 2010). Although one of the causes of severe asthma with elevated MMP-12 levels can be attributed to viral infections such as RSV, MMP408 has potential as a therapeutic agent for MMP-12–dependent neutrophilic asthma induced by both RSV and non-RSV infection. Incidentally, targeting IFN-β might suppress MMP-12 for reasons discussed in the next paragraph, similarly as treatment with MMP408. However, targeting IFN-β might be inappropriate because it could counteract the antiviral effect. For similar reasons, the pathway involving IFN-β, the IFN-β–induced upregulation of IL-4Rα expression, and MMP-12 production has not been directly described *in vivo*. Instead, by combining *in vivo* and *in vitro* models, we described a pathway from RSV infection to MMP-12 upregulation.

RSV infection highly induces MMP-12 expression by M2-like macrophages, but not by non-activated macrophages. We and other research groups illustrated that at least STAT6 activation, which is observed in M2-like macrophages, is required for MMP-12 expression (Nelson et al., 2012; Trojanek et al., 2014; Weng et al., 2018). Further increases of MMP-12 expression are not attributable to increased IL-4 and IL-13 expression in HDM/RSV mice. Conversely, the upregulation of IL-4Rα induced by IFN-β during RSV infection evokes MMP-12. In naïve mice, RSV infection induces both the IFN-β/IL-4Rα axis and IL-4/IL-13 production, leading to the induction of M2-like macrophages (Shirey et al., 2010). The appearance of these M2 macrophages plays a role in the resolution of inflammation and tissue repair in this model. Contrarily, RSV infection in mice during allergic airway inflammation induced macrophages similarly as observed for macrophages in naïve mice, but macrophages in our model induced substantial MMP-12 production and exacerbated airway inflammation. Because macrophages that only moderately express IL-4Rα in naïve mice are probably not strongly stimulated by high concentrations of IL-4/IL-13, these macrophages might be defined as normal M2-like macrophages. By contrast, macrophages that highly express IL-4Rα overexpress MMP-12 upon stimulation with higher levels of Th2 cytokines during allergic airway inflammation. We named them hyper M2-like macrophages. Although it is difficult to define the threshold of change from normal M2-like macrophages to hyper M2-like macrophages that highly express MMP-12, it is obvious that the change requires IFN-β and IL-4Rα expression above certain levels. Targeting only hyper M2-like macrophages, not including normal M2-like ones, might not only prove direct involvement in exacerbations by hyper M2-like macrophages in our model, but also treat exacerbations associated with overproduction of MMP-12.

In conclusion, our study also revealed that MMP-12 produced by RSV-induced hyper M2-like alveolar macrophages exacerbates airway inflammation with neutrophil infiltration. Therefore, the development of therapeutic agents targeting MMP-12 instead of inhaled steroids targeting eosinophils might provide a breakthrough treatment for the exacerbation of asthma or neutrophilic asthma.

### Limitation of the study

Our findings revealed one mechanism by which RSV infection exacerbates allergic airway inflammation. Future studies using a large number of clinical samples from patients with asthma and RSV infection are needed to assess whether the results obtained in the mouse model can be extrapolated to clinical samples. For example, measuring MMP-12 concentrations before and after exacerbation in patients with asthma will clarify whether MMP-12 is a true exacerbating factor.

## STAR★Methods

### Key resources table

**Table:** 

REAGENT or RESOURCE	SOURCE	IDENTIFIER
**Antibodies**		

anti-mouse Gr-1 antibody (Clone 1A8)	Bio X Cell	Cat# BE0075, RRID:AB_10312146
Rat IgG2a Ab (Clone 2A3)	Bio X Cell	Cat# BE0089, RRID:AB_1107769
rabbit anti-mouse MMP-12 polyclonal Ab	Santa Cruz Biotechnology	Cat# bs-1854R, RRID:AB_10856040
anti-mouse CD16/CD32 antibody	BD Pharmingen	Cat# 553142, RRID:AB_394657
anti-mouse CD45 antibody	BD Pharmingen	Cat# 560694, RRID:AB_1727492
anti-mouse F4/80 antibody	BD Pharmingen	Cat# 562127, RRID:AB_10893815
anti-mouse CD11c antibody	BD Pharmingen	Cat# 553801, RRID:AB_395060
anti-mouse CD64 antibody	BD Pharmingen	Cat# 139307, RRID:AB_2561962
anti-mouse Siglec-F antibody	BD Pharmingen	Cat# 562068, RRID:AB_10896143
anti-mouse MHC II antibody	BD Pharmingen	Cat# 557000, RRID:AB_396546
anti-mouse Ly6G antibody	BioLegend	Cat# 127605, RRID:AB_1236488
anti-mouse IL-4Rα antibody	BioLegend	Cat# 144807, RRID:AB_2750451
anti-mouse MMP-12 antibody	BioLegend	Cat# 17-7041-81, RRID:AB_469493
anti-mouse IL-17A antibody	BioLegend	Cat# 506915, RRID:AB_536017
anti-mouse phospho-Stat6 antibody	CST	Cat# 56554, RRID:AB_2799514
anti-mouse Stat6 antibody	CST	Cat# 5397, RRID:AB_11220421

**Bacterial and virus strains**		

Respiratory syncytial virus (A2)	Originally provided by Dr. Stokes Peeble (Vanderbilt University)	N/A

**Chemicals, peptides, and recombinant proteins**		

House dust mite antigen	Greer Laboratories	XPB81D3A2.5
Dexamethasone	Sigma-Aldrich	D2915
MMP408 (MMP-12 inhibitor)	Merck	444291
Recombinant MMP-12	R&D systems	3467-MPB-020
Recombinant IL-4	R&D systems	404-ML-010
Recombinant IL-13	R&D systems	413-ML-005
Recombinant IFN-β	R&D systems	12400-1
AS1517499 (STAT6 inhibitor)	Sigma-Aldrich	1906-5MG
*Muc5b probe*	Applied Biosystems	Mm00466391_m1
*Il4* probe for RT-PCR	Applied Biosystems	Mm00445259_m1
*Il5* probe for RT-PCR	Applied Biosystems	Mm00439646_m1
*Il13* probe for RT-PCR	Applied Biosystems	Mm00434204_m1
*Mmp12* probe for RT-PCR	Applied Biosystems	Mm00500554_m1
*Cxcl1* probe for RT-PCR	Applied Biosystems	Mm04207460_m1
*Cxcl2* probe for RT-PCR	Applied Biosystems	Mm00436450_m1
*Il17a* probe for RT-PCR	Applied Biosystems	Mm00439618_m1
*Arg1* probe for RT-PCR	Applied Biosystems	Mm00475988_m1
*Ifnb* probe for RT-PCR	Applied Biosystems	Mm00439552_s1
*Il4ra* probe for RT-PCR	Applied Biosystems	Mm00439635_m1
*Gapdh* for RT-PCR	Applied Biosystems	Mm99999915_g1

**Critical commercial assays**		

Mouse IL-4 DuoSet ELISA	R&D	DY404-05
Mouse IL-5 DuoSet ELISA	R&D	DY405-05
Mouse IL-13 DuoSet ELISA	R&D	DY413-05
Mouse MMP-12 ELISA	abcam	ab213878
Cell and tissue-staining Rabbit Kit (DAB)	R&D	CTS005
Cytofix/Cytoperm kit	BD Pharmingen	554714

**Experimental models: cell lines**		

RAW264.7	ATCC	TIB-71™
MLE-12	ATCC	CRL-2110™

**Experimental models: organisms/strains**		

Mouse: C57BL/6J (B6)	Japan SLC, Inc.	N/A
Mouse: B6.129X-Mmp12tm1Sds/J (MME KO: MMP-12 KO)	The Jackson Laboratory	N/A

**Software and algorithms**		

FlowJo software package (version 9)	TreeStar	N/A
ImageJ software (version 1.52a)	NIH	https://imagej.nih.gov/ij/
Prism 9	GraphPad Software	N/A

### Resource availability

#### Lead contact

Further information and requests for resources and reagents should be directed to and will be fulfilled by the Lead Contact, Takehiko Shibata (tshibata@tokyo-med.ac.jp).

#### Materials availability

This study did not generate new unique reagents.

#### Data code and availability

• All data are available from the Lead Contact on request.
• This study did not generate/analyze any code.
• Any additional information required to reanalyze the data reported in this paper is available from the lead contact upon request.

### Experimental models and subject details

#### Mice

C57BL/6 male mice (6 weeks old) were obtained from Japan SLC, Inc. (Hamamatsu, Japan), and matrix metalloproteinase 12 (MMP-12)–null mice (6 weeks old) were purchased from The Jackson Laboratory (Bar Harbor, ME, USA). Experimental protocols were approved by the Animal Experiment Committee of the National Institute of Infectious Diseases and Tokyo Medical University.

#### Experimental model of RSV-induced exacerbation of allergic airway inflammation

Mice were initially sensitized with 10 μg of house dust mite antigen (HDM; Greer Laboratories, Lenoir, NC, US) or PBS (control) via intranasal (i.n.) injections. One week later, mice were challenged with 1 μg of HDM or PBS via daily i.n. injections for 5 days (designated the HDM group). Three days later, these mice were infected with RSV (1 × 10^6^ PFU) via i.n. injections. All mice were assessed on day 4 after RSV infection (designated the HDM/RSV group).

#### Neutrophil depletion in HDM/RSV mice

Two days after the last HDM injection, mice were injected with 50 μg of anti-mouse Gr-1 antibody (Ab, 1A8; Bio X Cell, NH, USA) or Rat IgG2a Ab (2A3; Bio X Cell) via intraperitoneal (i.p.) injections. Two hours later, mice were infected with RSV (1 × 10^6^ PFU) via i.n. injections. These mice were treated with anti-mouse Gr-1 Ab or rat IgG2a Ab via i.p. injections every 24 h after RSV infection. All mice were assessed on day 4 after RSV infection.

#### Treatment of HDM/RSV mice with dexamethasone

Four days after the last HDM injection, mice were injected with 250 μg of dexamethasone (Sigma-Aldrich, Saint Louis, MO, USA) or PBS via i.p. injections. Two hours later, mice were infected with RSV (1 × 10^6^ PFU) via i.n. injections. These mice were treated with dexamethasone or PBS via i.p. injections every 24 h after RSV infection. All mice were assessed on day 4 after RSV infection.

#### Treatment with an MMP-12 inhibitor or recombinant MMP-12 (rMMP-12)

Four days after the last HDM injection in the HDM group, mice were injected with 100 μg of the selective MMP-12 inhibitor MMP408 (Merck, Darmstadt, Germany) or hydroxypropyl methylcellulose (HPMC) via gastrointestinal (g.i.) injections. Two hours later, mice were infected with RSV (1 × 10^6^ PFU) via i.n. injections. Mice were treated with MMP408 or HPMC via g.i. injections every 24 h after RSV infection. All mice were assessed on day 4 after RSV infection. In a separate experiment, 5 days after the last HDM injection in the HDM group (2 days after RSV infection), mice were treated with rMMP-12 (1 μg/30 μl; R&D systems) or PBS via i.n. injections every 24 h. All mice were assessed on day 4 after RSV infection.

#### Measurement of AHR

Respiratory system resistance (Rrs), elastance (Ers), Newtonian airway resistance (Rn), tissue damping (G), tissue elastance (H), and compliance (Crs) were assessed using flexiVent (SCIREQ, Montreal, Canada) and Rrs was shown as AHR in all groups of mice. Once the baseline airway resistance was established, increasing doses (i.e., 6.25, 12.5, 25, or 50 mg/ml in saline) of methacholine (Wako Pure Chemical Industries, Ltd., Osaka, Japan) were administered with aerosol, and AHR was monitored. Whole-lung lobes from each mouse were dissected and snap-frozen for cellular and proteomic analyzes or fixed in 10% formalin for histological analysis. Sodium pentobarbital (0.04 μg/kg body weight; Kyoritsu Seiyaku Corporation, Tokyo, Japan) was used to anesthetize mice before intubation and ventilation.

#### Treatment of macrophages and lung epithelial cells *in vitro*

RAW264.7 macrophages (American Type Culture Collection (ATCC), Manassas, VA, USA) were stimulated with IL-4 (10 ng/ml) and IL-13 (10 ng/ml) for 24 h at 37°C and followed by with or without IFN-β stimulation (5, 50 U/ml) for 24 h at 37°C. In some experiments, IL-4/IL-13 stimulated macrophages were treated with AS1517499 (1 μg/ml), which is a STAT6 inhibitor, for 24 h at 37°C. In a separate experiment, MLE-12 cells (ATCC), which are mouse lung epithelial cells, were stimulated with IFN-β (0.01, 0.1, 1 μg/ml) for 24 h at 37°C.

### Methods details

#### RNA extraction, RT-PCR, and quantitative PCR

Total RNA was isolated from the whole-lung tissue or cultured cells using TRIzol reagent (Invitrogen/Life Technologies, Carlsbad, CA, USA). The purified RNA was treated with DNase I and 20 μg of RNA was reverse-transcribed into cDNA using TaqMan reverse transcription reagents (Applied Biosystems, Foster City, CA, USA). The transcript levels of *Muc5b*, *Il4*, *Il5*, *Il3*, *Mmp12*, *Cxcl1*, *Cxcl2*, *Il17a*, *Arg1*, *Ifnb*, *Il4ra*, and *Rsv-f* were determined using RT-PCR. The fold difference in the expression levels was calculated using the ΔΔCt method, following the manufacturer's instructions (Applied Biosystems). GAPDH was used as an internal control. The fold-changes in expression were calculated by comparing the target gene expression in the experimental group with that in the control mice and macrophages, which was assigned a value of 1.

#### Determination of Th2 cytokine and MMP-12 levels

The levels of IL-4, IL-5, and IL-13 in the whole-lung were determined using a standardized sandwich ELISA (R&D Systems), following the manufacturer's instructions. The recombinant murine proteins (R&D Systems) were used to generate the standard curves. The ELISA detection limits for the cytokines were as follows: IL-4, 15.6 pg/mL; IL-5, 31.3 pg/mL; IL-13, 62.5 pg/mL. The mouse matrix metalloproteinase-12 (MMP-12) level in the bronchoalveolar lavage fluid and supernatant of alveolar macrophages *ex vivo* were determined using standardized sandwich ELISA (abcam, Cambridge, UK), following the manufacturer's instructions. The ELISA detection limit was consistently >62.5 pg/mL. All reactions were stopped by adding 25 μL of 1 M H_2_SO_4_. The sample absorbance was measured at 450 nm using a Model 680 microplate reader (BioRad, Hercules, CA).

#### Whole-lung immunohistochemical and histological analyses

The whole lungs were fully inflated with 10% formalin, dissected, and placed in fresh 10% formalin at room temperature for 24 h. Routine histological techniques were used to embed the tissue in paraffin. Fixed lung sections were stained with Hematoxylin-Eosin (HE) and Periodic Acid-Schiff (PAS). The specific proteins were detected using the rabbit anti-mouse MMP-12 polyclonal Ab (Santa Cruz Biotechnology; SC22759, 1:100). Other tissue sections were incubated with the control IgG isotype Ab. The slides were developed using the mouse HRP-(3,3′-diaminobenzidine) (DAB) cell and tissue-staining kit (R&D Systems), following the manufacturer's instructions. Additionally, the tissue sections were subjected to H&E staining. The slides were visualized under a light microscope (Nikon TE2000, Tokyo, Japan), and the images were captured using a camera attached to the microscope.

#### Flow cytometry analysis

The whole-lung samples were obtained from all groups of mice on day 4 post-RSV challenge and were incubated with collagenase type I (1 mg/mL; Thermo Fisher Scientific, Waltham, MA) and DNase I (1 μg/mL; Sigma-Aldrich, Saint Louis, MO) at 37°C for 45 min. The whole-lung cell suspensions were then incubated with the anti-CD16/CD32 (2.4G2, BD Pharmingen; 553142, 1:200) antibodies, followed by incubation with the fluorescent dye-mAb conjugates: anti-CD45 (BD Pharmingen, San Jose, CA; 560694, 1:200), anti-F4/80 (BD Pharmingen; 562127, 1:200), anti-CD11c (BD Pharmingen; 553801, 1:500), anti-CD64 (BD Pharmingen; 139307, 1:200), anti-Siglec-F (BD Pharmingen; 562068, 1:200), anti-MHC II (BD Pharmingen; 557000, 1:333), anti-Ly6G (BioLegend, CA; 141703, 1:160), anti-IL-4Rα (BioLegend; 144807, 1:160) for 15 min. For intracellular staining of the cytokines, the lung cells (10^6^ cells per well) were stimulated with phorbol myristate acetate (50 ng/mL) and ionomycin (1 μM) in the presence of GolgiStop (BD Cytofix/Cytoperm kit; BD Biosciences Pharmingen) at 37°C for 5 h. The cells were resuspended in the fixation/permeabilization solution (BD Cytofix/Cytoperm kit; BD Pharmingen) and stained with the anti-MMP-12 antibody (BioLegend; 17-7041-81, 1:100), anti-IL-17A antibody (BioLegend; 506915, 1:80), anti-IFN-γ antibody (BD Pharmingen; 554411, 1:100), and anti-IL-4 antibody (Thermo Fisher Scientific; 17-7041-81, 1:80) for 30 min. The data were acquired using a FACSCanto II machine and FACS Diva software 8.0 (BD Pharmingen). All data were analyzed using the FlowJo software package (TreeStar, Ashland, OR).

#### Western blotting

RAW264.7 macrophages were stimulated with IL-4 and IL-13 for 1h. The samples were lysed using a lysis buffer. The lysates were then resolved by sodium dodecyl sulfate gel electrophoresis (SDS-PAGE). The resolved proteins were transferred to a polyvinylidene fluoride (PVDF) membrane using iBlot (Thermo Fisher Scientific). The membrane was then incubated with 5% skimmed milk prepared in Tris-buffered saline containing 0.1% Tween 20 at room temperature for 1 h. The membrane was incubated with the rabbit anti-phospho-Stat6 (CST, 56554S, 1:2000), rabbit anti-Stat6 (CST, 5397S, 1:2000) for 1 h at room temperature. The membrane was washed and incubated with the horseradish peroxidase (HRP)-conjugated goat anti-rabbit anti-IgG (Abcam, ab6721, 1:2000) or HRP-conjugated goat anti-mouse anti-IgG (Cell Signaling Technology, Tokyo, Japan, 7076s, 1:1000) antibodies. Next, the membrane was incubated with the enhanced chemiluminescence (ECL) western blotting detection reagents (Amersham Biosciences, UK) for 1 min, following the manufacturer's instructions. The protein bands were analyzed using ImageQuant LAS-4000 and dark box (FujiFilm, Tokyo, Japan). To determine the phospho-Stat6 level to total Stat6 ratio, the immunoreactive band were quantified using the ImageJ software version 1.52a (National Institutes of Health, Bethesda, MD, USA).

#### DNA microarray analysis

DNA microarray analysis was undertaken by Filgen (Nagoya, Japan) using a ClariomTM S Assay Mouse (Thermo Fisher Scientific). The data were analyzed by software from Filgen.

### Quantification and statistical analysis

All data were analyzed using GraphPad Prism Software 7 and 9 (GraphPad Software, La Jolla, CA, USA) and presented as the mean ± SEM. In addition, a *t*-test or ANOVA was used to assess differences between groups, and P ˂ 0.05 was regarded as statistically significant.
