# Supplementary material for: RSV infection-elicited high MMP-12–producing macrophages exacerbate allergic airway inflammation with neutrophil infiltration
Source: iScience. 2021 Oct 2;24(10):103201. doi: 10.1016/j.isci.2021.103201 (PMC8524145; doi:10.1016/j.isci.2021.103201)
Supplement: Document S1. Figures S1–S3 [file mmc1.pdf]

iScience, Volume *24*

Supplemental information

RSV infection-elicited high MMP-12–producing macrophages exacerbate allergic airway inflammation with neutrophil infiltration

Airi Makino, Takehiko Shibata, Mashiro Nagayasu, Ikuo Hosoya, Toshiyo Nishimura, Chihiro Nakano, Kisaburo Nagata, Toshihiro Ito, Yoshimasa Takahashi, and Shigeki Nakamura

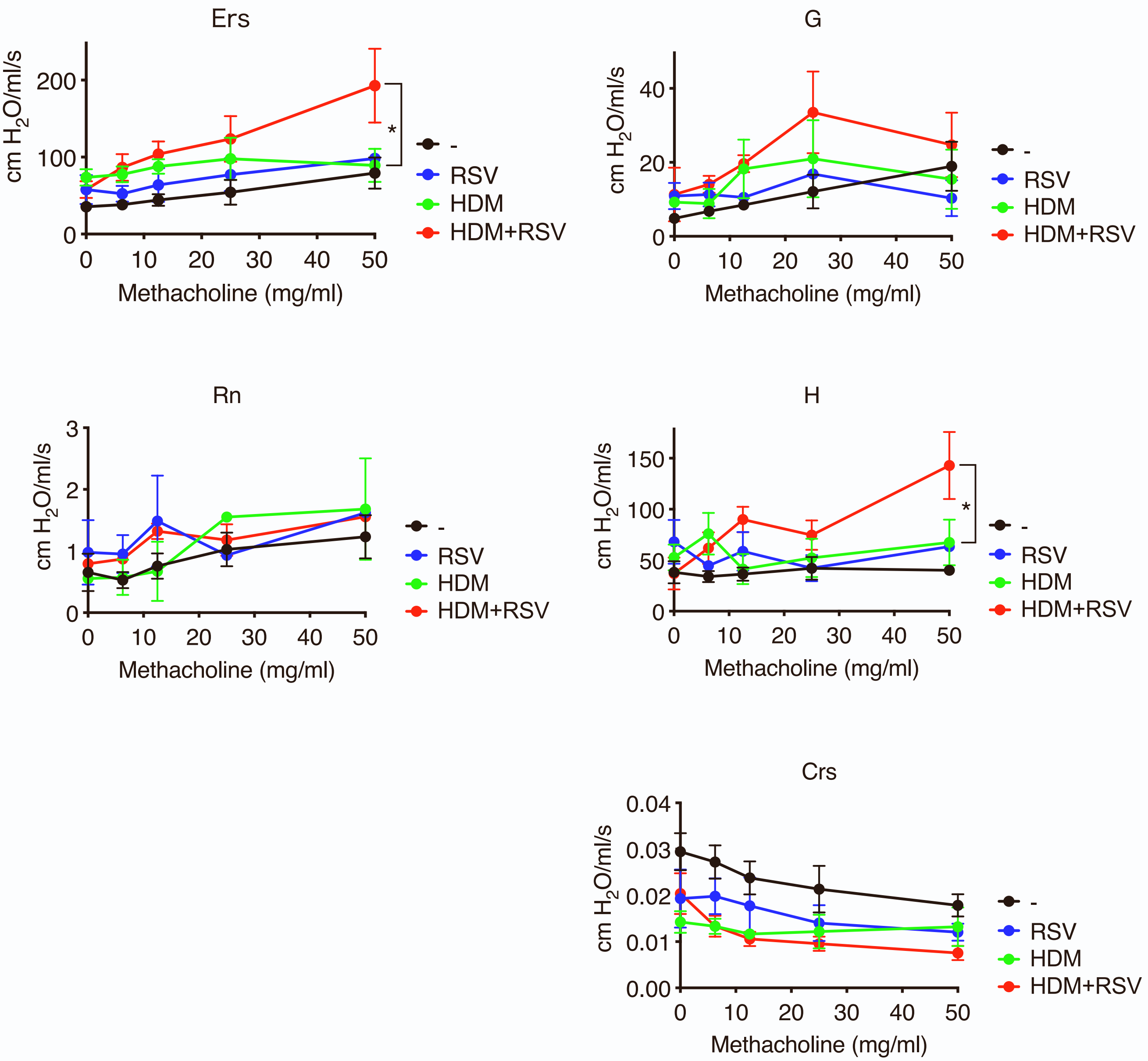

Figure S1. Airway responsiveness to inhaled methacholine in allergic airway inflammation after respiratory syncytial virus (RSV) infection. Related Figure 1B
Measurement of elastance (Ers), Newtonian airway resistance (Rn), tissue damping (G), tissue elastance (H), and compliance (Crs) in vehicle-injected mice (–: control), RSV-infected mice (RSV), HDM-sensitized mice (HDM), and RSV-infected HDM-sensitized mice (HDM/RSV).
The data are expressed as the mean ± SEM (n = 5-6). *$P < 0.05$.

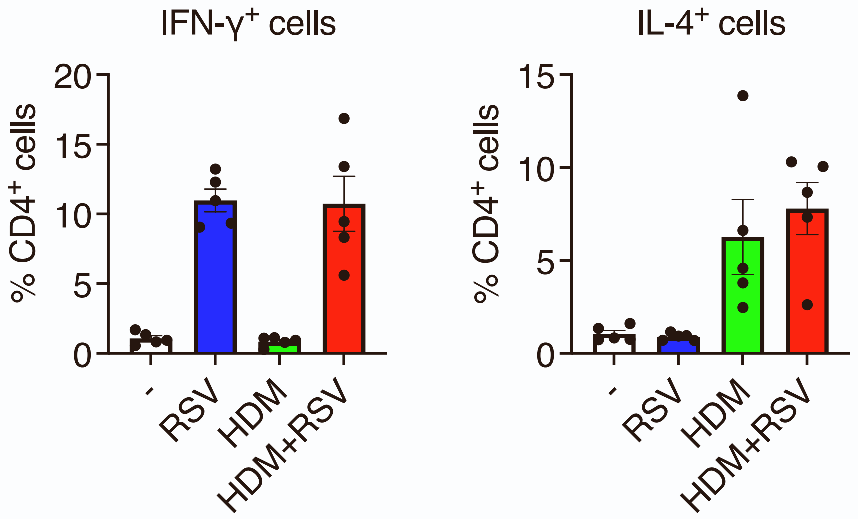

Figure S2. Th1 and Th2 cells do not increase in exacerbation of allergic airway inflammation associated with RSV infection. Related Figure 4H.

Cells generated from the lungs of vehicle-injected mice (–: control), HDM, RSV, and HDM/RSV groups were stimulated with PMA and Ionomycin and then stained for IFN-γ and IL-4. A percentage of IFN-γ^+ cells (A) and IL-4^+ cells (B) in CD4^+ T cells.

The data are expressed as the mean ± SEM (n = 5).

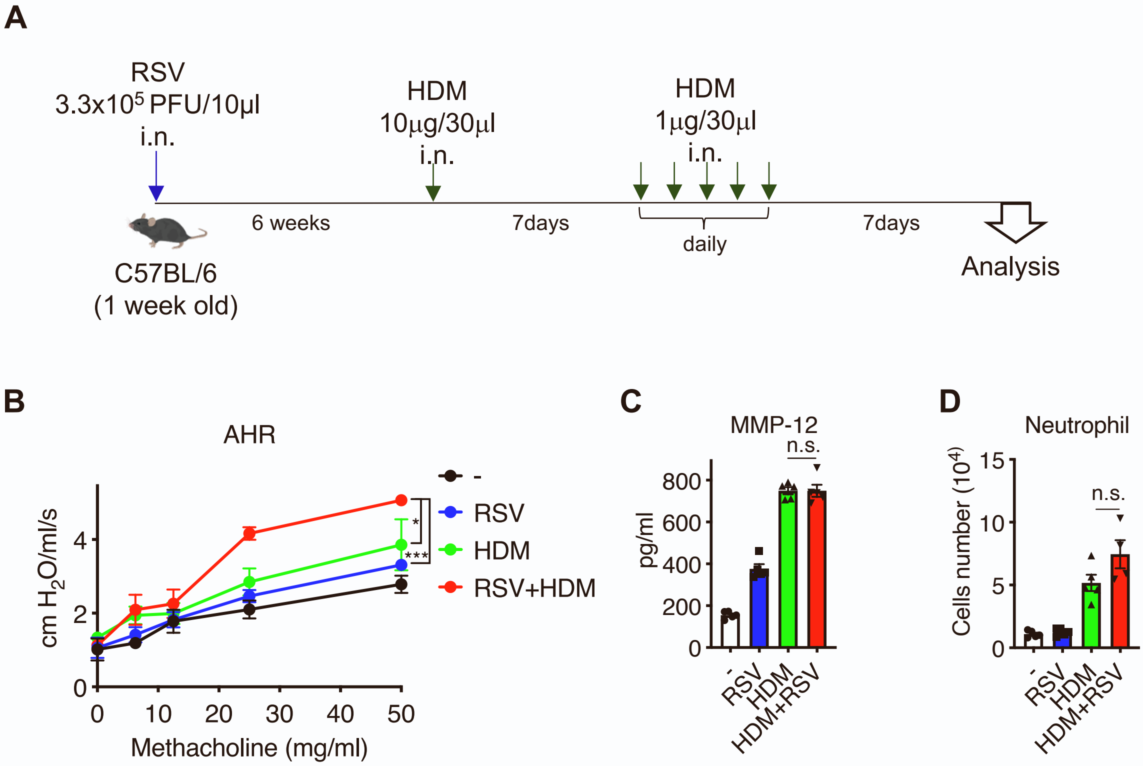

Figure S3. MMP-12/neutrophil axis is not involved in increased airway resistance in HDM exposure after RSV infection. Related to Figure 7.

(A) Mice were infected with RSV prior to house dust mite antigen (HDM) exposure.

(B) Measurement of respiratory system resistance (Rrs) for airway hyperresponsiveness (AHR) in vehicle-injected mice (–: control), RSV-infected mice (RSV), HDM-sensitized mice (HDM), and HDM-sensitized RSV-infected mice (RSV/HDM).

(C) Protein level of MMP-12 in whole lungs in the control, HDM, RSV, and HDM/RSV groups.

(D) The number of neutrophils in the control, HDM, RSV, and HDM/RSV groups.

The data are expressed as the mean ± SEM (n = 4–5). $^*P < 0.05$, $^{***}P < 0.001$.
